# Differences in inflammatory markers, mitochondrial function, and synaptic proteins in male and female Alzheimer's disease *post mortem* brains

**DOI:** 10.1002/alz.70645

**Published:** 2025-10-01

**Authors:** Alex J. T. Yang, Ahmad Mohammad, Robert W. E. Crozier, Lucas Maddalena, Evangelia Tsiani, Adam, J. MacNeil, Gaynor E. Spencer, Aleksandar Necakov, Paula Duarte‐Guterman, Jeffery Stuart, Rebecca E. K. MacPherson

**Affiliations:** ^1^ Department of Health Sciences Brock University St. Catharines Ontario Canada; ^2^ Department of Biological Sciences Brock University St. Catharines Ontario Canada; ^3^ Centre for Neuroscience Brock University St. Catharines Ontario Canada; ^4^ Department of Psychology Brock University St. Catharines Ontario Canada

**Keywords:** Alzheimer's disease, brain metabolism, human brain, inflammation, mitochondria function, sex differences

## Abstract

**INTRODUCTION:**

Inflammation and mitochondrial impairments are suggested to underlie Alzheimer's disease (AD) development. This study examined whether metabolic, synaptic, and inflammatory markers in AD differed from non‐demented brains.

**METHODS:**

Male and female AD brains were analyzed by immunofluorescence, Western blot, enzyme‐linked immunosorbent assay–based cytokine, and mitochondrial respiration analysis.

**RESULTS:**

AD brains had greater Akt phosphorylation, but only AD males had greater downstream mammalian target of rapamycin phosphorylation. AD females showed lower mitochondrial complex IV respiration. AD brains had greater expression of synaptic markers α‐Amino‐3‐hydroxy‐5‐methyl‐4‐isoxazolepropionic acid, glutamate receptor 1, and synaptophysin, while AD females had a higher expression ELKS1. Microglial expression was lower in AD gray matter, AD females had higher microglial expression in white matter, while cytokine interleukin 2 content was greater in AD brains.

**DISCUSSION:**

Markers of impaired insulin signaling, impaired mitochondrial function, and greater neuroinflammation were found in AD brains. Female brains had greater differences in metabolic signaling than males and this dysregulation is unique/worse with AD.

**Highlights:**

- Neuroinflammation and metabolic function are worse with Alzheimer's disease (AD).
- Female brains exhibit more distinct changes in metabolic signaling than males.
- Female brains have worse metabolic changes with AD.
- Harmful inflammatory and mitochondrial signaling may promote AD.

## 1 BACKGROUND

Recent developments in Alzheimer's disease (AD) research highlight the impact that metabolic dysregulation has on brain health and the development of AD.^1^ Specifically, neuroinflammation and mitochondrial impairments, driven by metabolic dysfunction, are suggested to be detrimental to synaptic function.^2^, ^3^ Additionally, most literature relies on male models of AD despite female sex being the second greatest risk factor for late‐onset AD, second only to the aging process itself.^4^ Closing the gap related to sex differences in the literature is paramount for not only understanding AD, but providing accurate therapeutic treatment.

Synaptic transmission is an energy‐intensive process.^5^ Adenosine triphosphate (ATP) is required for every aspect of transmission which occurs at a rapid pace.^5^ Additionally, the substantial glucose use and prominent localization of mitochondria at synapses suggests significant metabolic involvement in synaptic transmission,^5^ highlighting that mitochondrial impairment could directly impact synaptic and cognitive function. Impairments in mitochondrial function have been reported in cell and animal AD models^6^ as well as human AD brains.^6^, ^7^, ^8^ Specifically, an early report by Parker and Parks^7^ found significant reductions in activity of isolated mitochondrial respiratory complex protein kinetics from brains of patients with AD.^7^ These results have been repeated by which impaired electron transport chain (ETC) flux through all respiratory complexes (CI‐IV)^1^, ^8^ as well as reduced respiratory complex protein content^8^ have been observed in AD brains. Impairments in mitochondrial membrane potential and mitochondrial DNA damage also directly alter both amyloid precursor protein (APP) processing and amyloid beta (Aβ) plaque burden,^8^, ^9^ indicating a relationship between mitochondrial impairments and AD pathology. Additionally, several metabolic proteins are reduced in content and/or activity with AD brains and correlate with greater Aβ deposits, such as impairments in insulin‐driven Akt/mammalian target of rapamycin (mTOR) complex 1 (mTORC1) signaling^10^, ^11^ and reductions in the enzyme activity of the major Kreb‐cycle enzymes, α‐ketoglutarate‐dehydrogenase and pyruvate dehydrogenase.^12^ Importantly, sex was not considered in the above‐mentioned studies.^1^, ^6^, ^7^, ^8^, ^9^, ^12^

The observed mitochondrial impairments may be a consequence of increased inflammation and activated microglia. Microglia are involved in the pruning of damaged synapses and other cellular debris such as Aβ peptides^2^ in response to pro‐inflammatory stimuli such as cytokine release. Activated microglia are also directly associated with driving pro‐inflammatory cytokine release (such as interleukin [IL]‐6, IL‐1β, and tumor necrosis factor alpha [TNF‐α]), as well as downstream inflammatory signaling^13^, ^14^ as part of their immune function. Importantly, the development of neuroinflammation and the chronic activation of microglia has been strongly linked to mitochondrial damage,^2^, ^15^ synaptic damage,^2^, ^16^, ^17^, ^18^ and AD progression.^2^ However, despite evidence pointing to a significant role of inflammation impacting mitochondrial function,^15^ these results have rarely been reported in humans.^14^

Prior to menopause, females experience protection from age‐related diseases including cardiovascular disease, type 2 diabetes, and AD.^19^ The loss of estrogen that occurs with menopause is thought to describe the difference in AD prevalence between sexes:^20^ significantly impaired reference memory and object recognition,^21^ dementia‐like behaviors, and neuronal loss in both the prefrontal cortex (PFC) and hippocampus have been reported in ovariectomized (OVX) rhesus monkeys,^22^ outcomes which estrogen supplementation does improve.^22^ Aβ processing is also increased post‐menopause (likely through estrogen loss)^23^ alongside an increased and rapid development of metabolic disease suggesting a metabolic role for estrogen.^19^ In support of its metabolic role, estrogen receptor‐β (ERβ) is highly expressed in brain mitochondria^24^ highlighting a distinct mitochondrial sensitivity to estrogen. When associated with impairments in mitochondrial morphology reported in OVX rhesus monkeys,^22^ impairments in female brain metabolism point to mechanisms that might be estrogen dependent.^24^ Additional evidence also points to aged females having a stronger activation of neuroinflammatory responses and greater microglial impairment,^25^ but this has not yet been confirmed in humans. Despite recent work describing sex differences in AD progression, further examination is required with respect to both mitochondrial function and/or neuroinflammatory differences.^26^

The current study aimed to determine whether, in *post mortem* brains of non‐demented (ND) and AD males and females, there are sex‐specific differences in (1) metabolic signaling, (2) synaptic protein content, and (3) microglial morphology and inflammatory cytokines.

## 2 METHODS

### 2.1 *Post mortem* human brain samples

This study was approved by the Brock University Research Ethics Board (Protocol reference #21‐219). Age‐matched male and female human brain samples were obtained from Brodmann area 10 of the PFC by The Douglas Bell Canada Brain Bank (Montreal, Quebec, Canada; http://douglasbrainbank.ca), which receives consent for *post mortem* brain tissue collection by donors or their legal representatives. These included male (*n* = 10; 78.6 ± 8.1 years of age) and female (*n* = 10, 79.4 ± 7.8 years of age) controls with no AD diagnosis (ND), and male (*n* = 10 male; 82.4 ± 5.9 years) and female (*n* = 10 female; 81.2 ± 7.0 years of age) samples with AD status. AD status was confirmed by *post mortem* histopathological analysis onsite by an affiliated neuropathologist of The Douglas Bell Canada Brain Bank. Diagnoses focused on the presence or absence of Aβ and tau tangle deposit, that was then compared to patient information surrounding the onset of dementia‐like symptoms. No information was available regarding the donor's apolipoprotein E ε4 status. The average brain weight was 1184.8 ± 136.8 g for ND and 1081.6 ± 168.5 g for AD brains and no differences between males and females were observed. *Post mortem* delay (PMD) was an average of 13.42 ± 3.77 hours for ND and 15.39 ± 4.62 hours for AD samples. Subject and sample characteristics are summarized in Table 1.

**RESEARCH IN CONTEXT**

1. **Systematic review**: The literature was reviewed using traditional (e.g., PubMed) sources. Alzheimer's disease (AD) has been widely studied with respect to amyloid beta plaques, but recent publications describe the importance of metabolism and how metabolic dysregulation could drive AD progression. Additionally, despite women being at greater risk for AD development, sex has long been underdiscussed. These relevant studies are appropriately cited.
2. **Interpretation**: Our findings describe distinct differences in the metabolism of AD brains. Our findings also show that female brain metabolism is distinctly dysregulated with AD.
3. **Future directions**: The study outlines areas of brain metabolism in which future research may benefit AD. These include (1) the role of microglia on synaptic health, (2) the impact of mitochondrial function on synaptic and cognitive outcomes, (3) sex differences on fuel uptake into the brain, and (4) the relationship between compensatory responses in the brain and AD progression between sexes.

**TABLE 1:** Demographics.

Group	Age years (SD)	Sex (M/F)	PMI hours (SD)	Brain weight grams (SD)
ND	79.00 (7.71)	10/10	13.42 (3.77)	1184.8 (136.8)
AD	81.80 (6.31)	10/10	15.39 (4.62)	1081.6 (168.5)

*Note*: ND versus AD; age *p =* 0.2164; PMI *p* = 0.8427; brain weight *p* = 0.1152.

Abbreviations: AD, Alzheimer's disease; ND, non‐demented; PMI, *post mortem* interval; SD, standard deviation.

### 2.2 Mitochondrial respiration

Respirometry analysis of previously frozen tissue homogenates, along with the corresponding tissue homogenization, was based on the method described in Acin‐Perez et al.,^27^ and Osto et al.,^28^ as well as reported by Troutwine et al.^8^

### 2.3 Tissue homogenization

Tissue samples were weighed and homogenized in ice‐cold mitochondrial assay solution (MAS; 1x, pH 7.4, consisting of 70 mM sucrose, 220 mM mannitol, 5 mM KH_2_PO_4_, 5 mM MgCL_2_, 1 mM EGTA, and 2 mM HEPES) at a ratio of 500 µL per 10 mg tissue. Homogenization was performed on ice using 20 strokes with a pre‐chilled glass‐glass Dounce homogenizer. After homogenization, samples were centrifuged at 2000 × g for 3 minutes at 4°C before the resulting supernatant (tissue homogenate) was either used immediately or stored at −80°C for later use. Protein concentrations were determined using the bicinchoninic acid (BCA) assay that included bovine serum albumin (BSA) standards prepared in homogenization buffer.

### 2.4 Respirometry assay

Respirometry assays were performed using an Agilent Seahorse XFe24 Analyzer. Tissue homogenates (10 µg) loaded into Seahorse XFe24 microplate wells in 60 µL of MAS (non‐ionic mannitol and sucrose‐based buffer; triplicate wells per sample) were then centrifuged at 2000 × g for 5 minutes with a low break setting (deceleration speed of 3/9 on Qiagen 4‐15C plate centrifuge) before the addition of 390 µL of prewarmed (37°C) MAS supplemented with cytochrome c (10 µg/mL, final) and alamethicin from *Trichoderma viride* (10 µg/mL, final) when the respiration buffer was added to each well. Compound injections containing all the tested substrates were prepared in MAS (75 µL injection per port): nicotinamide adenine dinucleotide + hydrogen (NADH; 1.43 mM, final), antimycin A (4 µM, final), N,N,N',N'‐tetramethyl‐p‐phenylenediamine dihydrochloride (TMPD) + ascorbate (0.55 mM and 1.11 mM, final, respectively), and sodium azide (50 mM, final). Mixing steps and measurement steps consisted of 30 seconds and 4 minutes, respectively. From the oxygen consumption rate (OCR) data, the following parameters were calculated: (1) respiratory capacity through Complex I = *OCR*_NADH_ − *OCR*_Antimycin A_, and (2) respiratory capacity through Complex IV = *OCR*_TMPD+Ascorbate_ − *OCR*_Azide_.

### 2.5 Western blot

PFC samples were homogenized in 20x lysis buffer per mg of wet brain weight (NP40 Cell Lysis Buffer; Life Technologies; CAT# FNN0021) supplemented with 34 𝜇L phenylmethylsulfonyl fluoride and 50 𝜇L protease inhibitor cocktail (Sigma; CAT# 7626‐5G, CAT# P274‐1BIL). Samples were then centrifuged at 10,000 g followed by sample lysate collection. A BCA assay was then performed to determine the protein concentration of the lysates. Samples were prepared (1 µg/µL) and equal amounts of protein were then electrophoretically separated on 10% sodium dodecyl sulfide polyacrylamide gel electrophoresis gels and transferred to nitrocellulose membranes (GE Life Science Ca. 10600002, 0.45 𝜇m). Membranes were blocked for 90 minutes at room temperature in 5% non‐fat dry milk‐TBST (tris‐buffered saline/0.1% tween 20). Membranes were then incubated in primary antibody diluted 1:1000 in 5% BSA–TBST overnight at 4°C with gentle agitation. The next day, membranes were incubated for 1 hour at room temperature with the appropriate secondary antibodies (1:2000; donkey antirabbit IgG [H+L], #711‐035‐152, Goat antimouse IgG [H+L], #115‐035‐003 Jackson ImmunoResearch) in 1% BSA–TBST. Membranes were rinsed three times for 5 minutes in TBST and proteins visualized by Western Lightning Plus‐ECL (PerkinElmer NEL103E001EA) using a ChemiDoc Imaging System (Bio‐Rad). After the fluorescence images were taken, the membranes were placed in Ponceau stain (Cat. No. PON002, BIOSHOP) for 10 minutes before being rinsed in distilled water and laid out to dry for imaging. The bands of protein were quantified and analyzed using AlphaView followed by a quantification of the Ponceau to ensure equal loading across the membrane. Band densitometry was quantified using Alpha Innotech software. Insulin receptor substrate 1 (IRS‐1 S636; Cell Signaling Technology #2388), total IRS‐1 (Cell Signaling Technology #2390), mTOR S2448 (Cell Signaling Technology #2971), total mTOR (Cell Signaling Technology #2972), Akt T308 (Cell Signaling Technology #9275), Akt S473 (Cell Signaling Technology #4058), total Akt (Cell Signaling Technology #4685), total P70S6K (Cell Signaling Technology #9202), and phosphorylated P70S6K (Santa Cruz SC‐11759) were measured as markers of insulin signaling. Synaptophysin (Cell Signaling Technology #5461), NeuN (Cell Signaling Technology #24307), mature brain‐derived neurotrophic factor (BDNF; Abcam ab108319), PSD‐95 (post‐synaptic marker, Cell Signaling Technology #36233), Homer‐1 (post‐synaptic marker, SC‐136358), synaptosomal‐associated protein of 25 kDa (SNAP‐25; Cell Signaling Technology #5304), and Vesicle‐associated membrane protein 2 (VAMP2; Cell Signaling Technology #13508) were measured as synaptic and neuronal markers. Peroxisome proliferator‐activated receptor gamma coactivator 1‐alpha and a total oxidative phosphorylation (OXPHOS) cocktail (ab110411)^8^ were measured as markers of mitochondrial synthesis and content, respectively.

### 2.6 Human inflammatory cytokine panel

IL‐1β, IL‐2, IL‐6, TNF‐α in PFC sample lysates were quantified via Simple Plex assay kits purchased from ProteinSimple (Bio‐Techne, #ST01E‐PS‐007487) and analyzed on an Ella next generation microfluidics platform (ProteinSimple) using Simple Plex software Runner and Explorer. Samples were loaded at a 1:2 dilution as per the manufacturer's recommendations. Results were normalized relative to protein concentration.

### 2.7 Immunohistochemistry

Formalin‐fixed PFC samples were sent to the Department of Biomedical Sciences at the University of Guelph for processing, sectioning (5 µm in a 1:10 series), and mounting. Bregma coordinates were not provided for these sections; instead, observed anatomical landmarks of the tissues were compared directly to those found in an atlas of human brain to confirm that the areas examined were identical between samples. Pre‐mounted paraffin sections were rehydrated following a modified deparaffinization protocol from Korzhevskii et al.^29^ Briefly, slides were washed in xylene (2x 10 minutes) followed by a rehydration in ethanol in decreasing concentration: 100% (2x 2 minutes), 95% (1x 2 minutes), 90% (1x 2 minutes), 70% (2x 2 minutes), and ddH_2_O (2x 2 minutes). Sections then underwent heat‐induced epitope retrieval and demasking in a sodium citrate buffer (10 mM Tri‐Sodium citrate dihydrate; pH = 6.0) in an 800 W microwave for 8 minutes.^29^, ^30^ After antigen retrieval, sections were outlined with a hydrophobic pen (abcam; ab2601) and prepared for antibody incubation. Sections were rinsed three times in 0.1 M phosphate‐buffered saline (PBS; pH; 7.4) between each step listed below unless otherwise stated. Sections were permeabilized and blocked with 3% normal goat serum (NGS) and 0.3% Triton‐X in PBS for 90 minutes before incubation with ionized calcium‐binding adaptor molecule 1 (Iba1; 1:1000, ab5076), ELKS1 (1:400, NBP1‐88177), and vesicular glutamate transporter 1 (vGLUT1; 1:400, MA5‐31373) primary antibodies for 48 hours at 4°C. After primary antibody incubation, sections were then incubated with the secondary antibody Alexafluor 594 (1:400; ab150132), Alexafluor 488 (1:400; ab150106), and Alexafluor 555 (1:400; ab150073) for 48 hours at 4°C. Sections were then incubated in 4',6‐diamidino‐2‐phenylindole (1:400) for 2 minutes before being counter stained with TrueBlack (Cell Signaling Technology 92401) for 5 minutes to inhibit autofluorescence. Slides were then allowed to fully dry before being cover‐slipped using polyvinyl alcohol mounting medium with DABCO.

### 2.8 Region of interest identification and analysis

A researcher blinded to experimental conditions imaged and analyzed Iba1+ cells, vGLUT, and ELK1 expression. Images were captured with the FluoView confocal microscope with 20x objectives. Visual analysis of PFC was performed between individuals and comparable regions of interest (ROIs) were defined. The distinct folds demarcating the differences between “white” and “gray” matter were selected as ROIs for the PFC as they represent the most consistently identifiable regions of the PFC between samples. *Z* stacks for each sample were captured with an *N* of two images taken per ROI per individual (*n* = 10 per sex and condition), where one *N *= one immunofluorescence (IF) section from an individual. IF analysis was conducted with ImageJ, where the number and morphology of microglia were quantified.^31^ The ImageJ particle analysis plugin was used to analyze total Iba1, vGLUT, and ELKS1 expression. After initial analysis, microglial morphology was assessed via the high‐throughput microglial analysis ImageJ plugin, MicrogliaMorphology, as described by Kim et al.^32^ Briefly, each Iba1 image was thresholded to identify and capture individual microglia with as many branches as possible connected to cell bodies that minimize overlap between cells. After this, individual microglial single‐cell images were generated in which the skeletonization of each single‐cell image generated data for eight morphological features per cell (maximum branch length, average branch length, numbers of end point voxels, junction voxels, triple points, branches, junctions, slab voxels, and quadruple points).^33^, ^34^ After skeletonization, fractal analysis was used to generate additional morphological measures (outlined in Figures S1, S2, and S3 in Supporting Information).^31^ Downstream microglial analysis was then conducted using the MicrogliaMorphology R plugin^32^ in which microglia were categorized into five major morphological states: ameboid, dystrophic, hypertrophic, ramified, and rod‐like.^32^ Morphological categorization was done by assessing extent of association of microglia with the 27 individual morphological markers that were then used to associate microglia with an individual morphology via hard clustering analysis (Figures S1, S2). Classification of microglial as either dystrophic (PC1; lowest branch thickness and length as explained by pixel density in hull, small cell bodies, and large number of branches and junctions), ameboid (PC2; lowest territory span, high circularity, and smallest branch lengths), rod‐like (PC3; greatest oblongness and lowest circularity), hypertrophic (PC4; average territory span and high branch thickness as explained by pixel density in hull), or ramified (PC5; largest territory span and branching complexity) were based on previous literature describing these microglial morphologies (Figure S3).^32^, ^35^, ^36^

### 2.9 Statistical analysis

For western blot and total fluorescence analysis, differences in protein content/phosphorylation status, and fluorescence levels were determined using two‐way analysis of variance (ANOVA) with sex and AD status as factors, followed by a Fisher least significant difference post hoc test. Differences in microglial morphological categorization based on sex and AD status were determined using three‐way ANOVA followed by a Bonferroni post hoc test. A value of *p* < 0.05 was considered significant, whereas *p* values between 0.051 and 0.055 were considered trending. For western blots, all data are reported as mean ± standard error of the mean with *N* values between 8 and 10 for all sample sets. Sample sets for IF analysis consisted of *N *= 8 to 10 for which one *N* consisted of two individual brain slices for each ROI pooled. Additionally, for morphological analysis of microglia, each individual ROI consisted of 1000 to 2000 individual microglial skeletons that were used to generate morphological data as described above.

## 3 RESULTS

### 3.1 Metabolic signaling

Metabolism plays an important role in maintaining both cognitive function^5^ and neuronal growth.^37^ As such, we investigated whether changes in metabolic signaling differed between cognitively normal and AD individuals. As dysregulated insulin signaling has been demonstrated in the progression of AD,^11^, ^38^ we investigated metabolic markers associated with insulin signaling and cell growth, Akt and mTORC1. Both Akt and mTORC1 are important kinases that are involved in a number of signaling cascades; however, they play an important role in insulin signaling. After insulin binding to its receptor, Akt (through S473 phosphorylation) activates mTORC1 (through the phosphorylation of mTOR S2448) and promotes protein and cell growth.^39^ The hyper‐phosphorylation of both Akt S473 and mTOR S2448 has also been identified in human AD samples and has been related to the blunting of insulin signaling through the serine phosphorylation of the IRS‐1.^11^ It is this IRS‐1 serine phosphorylation that is suggested to be a main driver in the brain insulin resistance seen with AD.^10^, ^40^ Because of their importance in both metabolism and AD, Akt and mTORC1 signaling represent an important pathway that allows for an examination of metabolic changes in AD.

**FIGURE 1:**
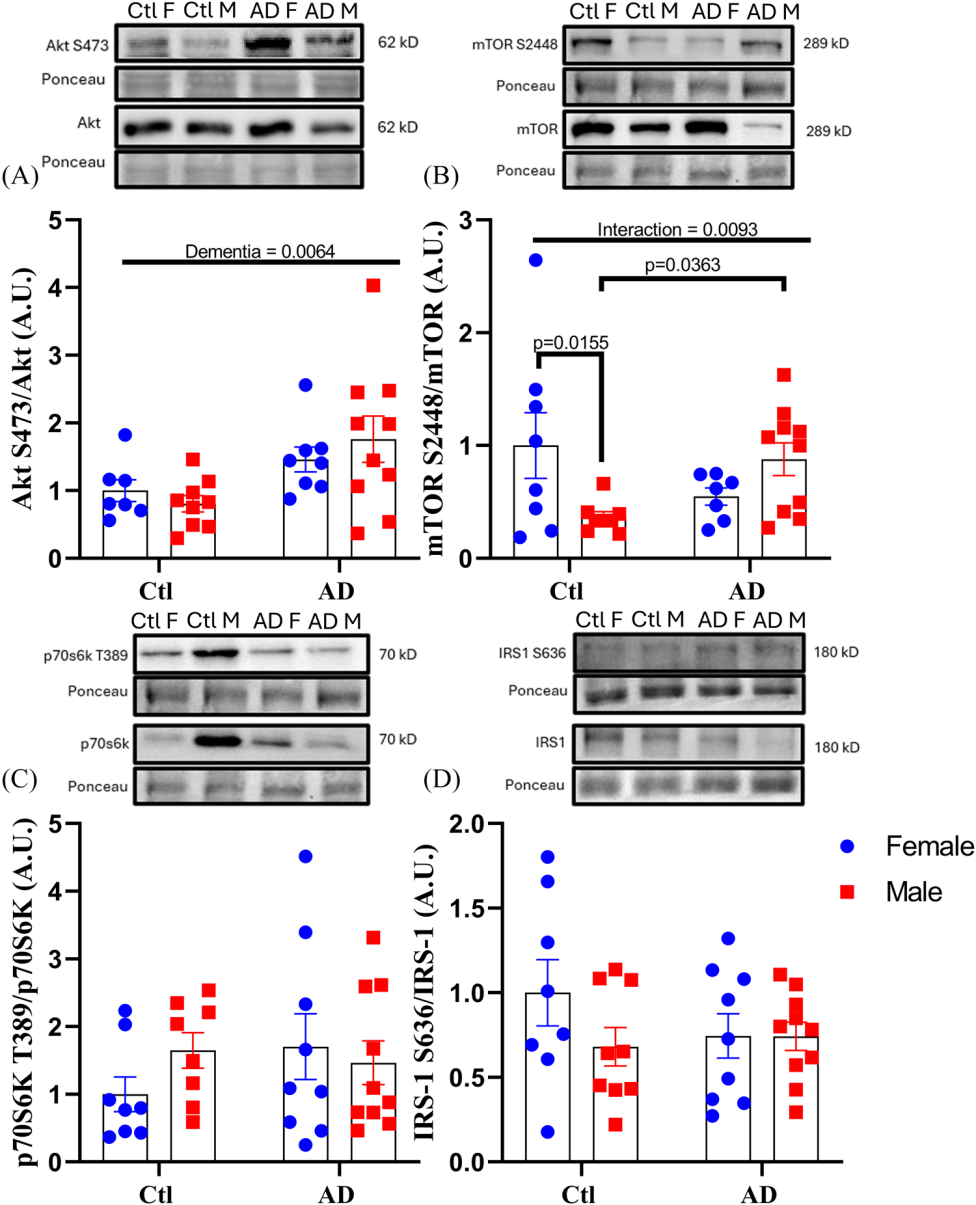
Akt and mTOR phosphorylation difference are seen with AD status and by sex. Changes in brain metabolic markers for PFC with and without AD. PFC; (A) AKT S473 phosphorylation, (B) mTOR S2448 phosphorylation, (C) p70S6K T389 phosphorylation, (D) IRS‐1 S636 phosphorylation. The same Ponceau is seen in (B) and (C) because the same membranes were probed for mTOR and p70S6K. Percent change represented as mean ± standard error of the mean. Differences between groups as determined using a two‐way analysis of variance followed by Fisher least significant difference post hoc analysis. AD, Alzheimer's disease; Ctl, control; mTOR, mammalian target of rapamycin; PFC, prefrontal cortex.

Within the PFC, Akt S473 phosphorylation (a marker of insulin‐specific Akt activation) was significantly higher in AD individuals with no impact of sex (Figure 1A; main effect of dementia, *F*[1, 30] = 8.580, *p* = 0.0064). This increase in Akt S473 phosphorylation in both AD males and females is consistent with previous reports that highlight significant upregulations in Akt phosphorylation in the brains of AD patients^10^ and would imply an increase in downstream Akt signaling such as on mTORC1. However, analysis of mTOR S2448 phosphorylation showed a significant two‐way interaction (Figure 1B; interaction, *F*[1, 29] = 7.766, *p* = 0.0093), whereby female controls had significantly higher mTOR S2448 phosphorylation than control males (Figure 1B; *p*_mTOR‐S2448 _= 0.0155) and AD males has significantly higher mTOR s2448 phosphorylation compared to control males (Figure 1B; *p*_mTOR‐S2448 _= 0.0363). There was no difference in mTOR S2448 phosphorylation between control and AD females (*p*_mTOR‐S2448 _= 0.0873) and no difference between AD females and AD males (*p*_mTOR‐S2448 _= 0.1841). These results would indicate that AD males exhibit greater mTOR S2448 phosphorylation that is consistent with greater Akt activity, but AD females do not. While previous reports have shown greater mTOR S2448 phosphorylation in *post mortem* human AD samples,^10^, ^11^ they did not examine differences by sex. Compared to the results reported here, changes in Akt and mTORC1 signaling would imply that greater Akt and mTOR phosphorylation is unique to males with AD. However, we observed no differences in the downstream markers of Akt and mTOR signaling, p70s6k T389 (interaction, *F*[1, 31] = 1.560, *p* = 0.210) or IRS‐1 S636 (interaction, *F*[1, 31] = 1.466, *p* = 0.2349) phosphorylation between any group (Figure 1C and D). These results imply that greater Akt S473 and mTOR S2448 phosphorylation do not result in an increase in the inhibitory phosphorylation of IRS‐1 S636. This is in direct contrast to previous reporting detailing the importance in the negative feedback loop of Akt‐mTORC1 on insulin signaling that was suggested to play a role in AD progression.^11^, ^40^

### 3.2 Mitochondrial respiratory capacity

While examining metabolic signaling pathways can elucidate some aspects of brain metabolism that might be changing with AD, examining changes in mitochondrial content and function allows for a more direct analysis of brain metabolism. To this end we analyzed the content of ETC proteins and OCR of the PFC mitochondria to explore differences in the metabolic state of the brain between males and females with and without AD.

**FIGURE 2:**
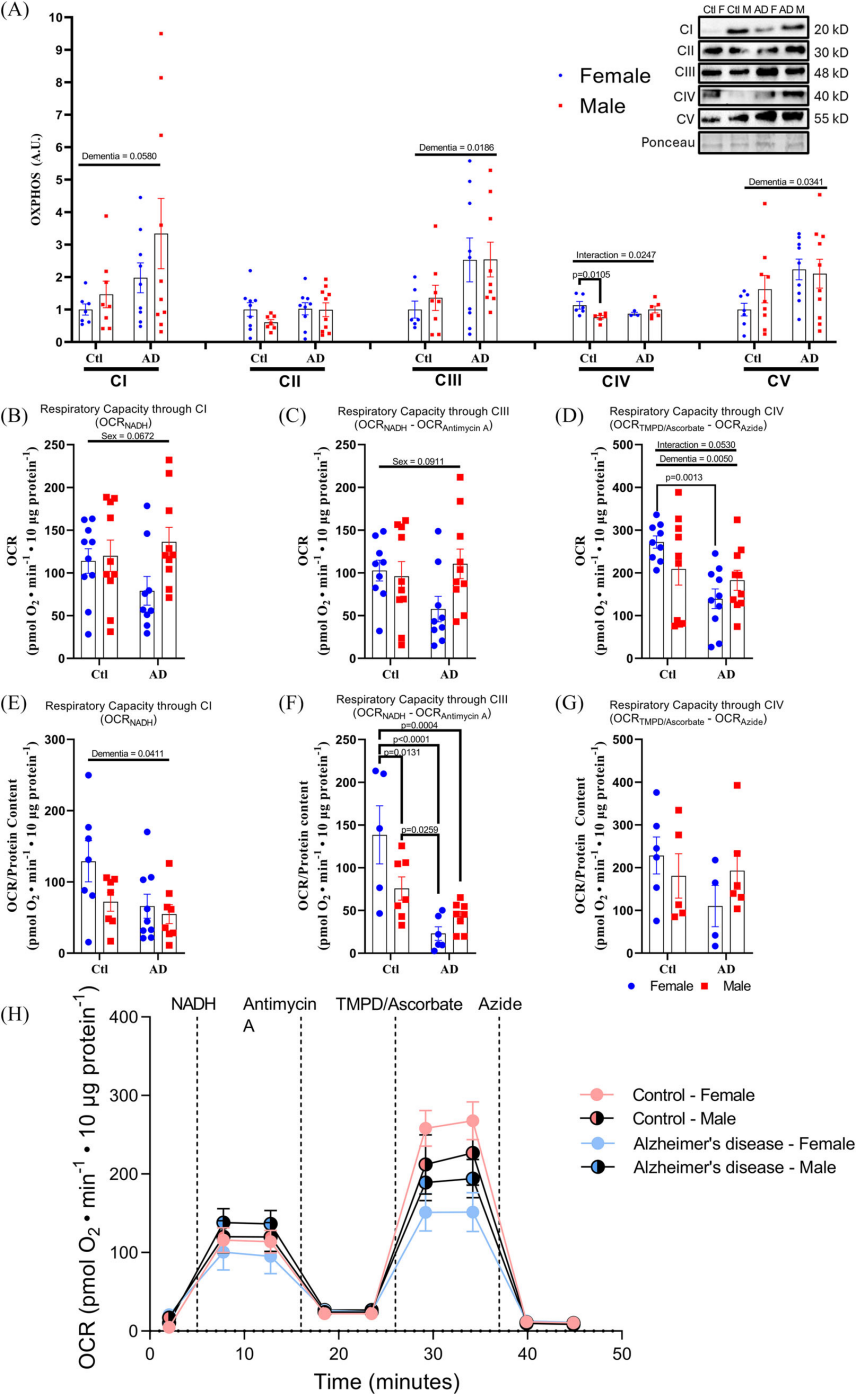
Mitochondrial respiration is impaired with AD despite increases in COX protein content. A,OXPHOS complex protein concentrations and oxygen consumption rates (OCR) in post mortem brain. (B)Quantification of OXPHOS protein content, OCR through complex I (NADH), (C) complex III (NADHwith antimycin A subtracted), and (D) complex IV (TMPD/Ascwith azide subtracted). (E),(F),and (G) OCR for complex I, III, and IV normalized to individual complex protein content. (H) representative tracing from the Seahorse XF Analyzer. n = 10 for Ctl females and Ctl males versus n = 10 for AD females and AD males. Proportional change (%) represented as mean ± standard error of the mean. Differences between groups as determined using a two‐way analysis of variance followed by Fisher least significant difference post hoc analysis. AD, Alzheimer's disease; COX, cyclooxygenase; Ctl, control; NADH, nicotinamide adenine dinucleotide + hydrogen;OCR, oxygen consumption rate; OXPHOS, oxidative phosphorylation; TMPD, N,N,N',N'‐tetramethyl‐p‐phenylenediamine dihydrochloride.

Examination of protein content of the mitochondrial complexes that form the ETC (complexes I–V) showed two‐way main effects for dementia status, whereby AD individuals had higher content for complex III (Figure 2A; dementia, *F*[1, 28] = 6.246, *p* = 0.0186) and complex V (Figure 2G; dementia, *F*[1, 31] = 4.916, *p* = 0.0341). Complex IV content showed a significant two‐way interaction (interaction, *F*[1, 17] = 6.067, *p* = 0.0247), whereby control males had significantly lower content than control females (Figure 2A; *p*_complex IV _= 0.0105). Complex I and complex II saw no difference in protein content between groups (Figure 2A). Overall, results from changes in ETC protein expression indicate that significant changes to mitochondrial machinery occur with AD that could reflect changes in mitochondrial function. Specifically, given their importance in driving H^+^ ions out of the inner mitochondrial membrane^41^ and driving ATP synthesis^42^ respectively, greater complex III and complex V expression may describe an increase in ETC function. As such, using previously established protocols,^8^, ^28^ a seahorse XF24 analyzer was then used to assess mitochondrial respiratory function. There was a main effect for dementia (*F* [1, 35] = 8.980 *p* = 0.0050) and a trending two‐way interaction (interaction, *F*[1, 35] = 4.011, *p* = 0.0530) whereby AD males and females had significantly lower OCR through complex IV (*p* = 0.0244 and *p* = 0.0013, respectively) compared to control females (Figure 2D). These data indicate that while increases in expression for ETC complexes were observed, a reduction in mitochondrial OCR occurs for both males and females with AD (Figure 2H). Additionally, though a trending significance only, the reduction in mitochondrial function with AD could primarily be driven by reductions in OCR seen in AD females, as no difference between control and AD males for complex IV OCR was observed. This would indicate that brains with AD pathology show greater mitochondrial dysfunction and that this dysfunction might be worse in female individuals with AD. However, because differences in ETC complex protein expression were seen, it is possible that changes in OCR are reflective of differences in the amount of protein present. As such, we normalized the OCR of complex I, III, and IV to their protein content to determine whether the differences observed in respiratory capacity were a result of greater/lower protein expression. After normalization, there were no differences in complex IV OCR between control and AD females (Figure 2G). Additionally, while no difference in complex I or complex III OCR were observed before normalization (Figure 2B, C), after normalization to total protein both complex I and III showed significant differences; complex I showed a main effect for dementia in which OCR was lower for both male and female AD individuals (Figure 2E; *p* = 0.0411) and complex III (Figure 2F) showed a significant interaction whereby control females exhibited significantly higher OCR compared to control males (*p* = 0.0131), AD females (*p* < 0.0001), and AD males (*p* = 0.0004). Importantly, complex III OCR was also different between control males and AD females (*p* = 0.0259) despite there being no differences between control and AD males. This relative OCR data with respect to protein content indicates that mitochondrial respiration rates are significantly reduced with AD with complex III OCR being significantly reduced in AD females and that increases in complex I and III protein content are likely a compensatory response to impairments in ETC function occurring with AD. Differences in mitochondrial respiration and ETC function between methods of analysis are not unusual; previous reports within *post mortem* human brain samples have noted discrepancies between methods of analysis. Specifically, differences between ETC flux assays such a Seahorse and Vmax‐assays have been reported by Wilkins et al. between complex II respiration in which Seahorse analysis showed no difference whereas the Vmax assay showed significant differences.^9^ A significant drawback of spectrophotometric assays is that they rely on supraphysiological electron donors and acceptors, yielding measurements that reflect non‐physiological maximal enzyme activity rather than physiological function. While this analysis is useful for determining enzymatic turn‐over and substrate responses, it is not as suitable to explore more nuanced respiratory changes the exist under physiological conditions such as those in the current study.

### 3.3 Synaptic and neuronal changes

AD, although characterized by synaptic damage and the death of neurons,^43^ has also been shown to have severe reductions in neuronal and synaptic protein content as the disease progresses.^44^, ^45^ However, the impact that sex might have on AD‐associated reductions in synaptic and neuronal proteins has not been explored.

**FIGURE 3:**
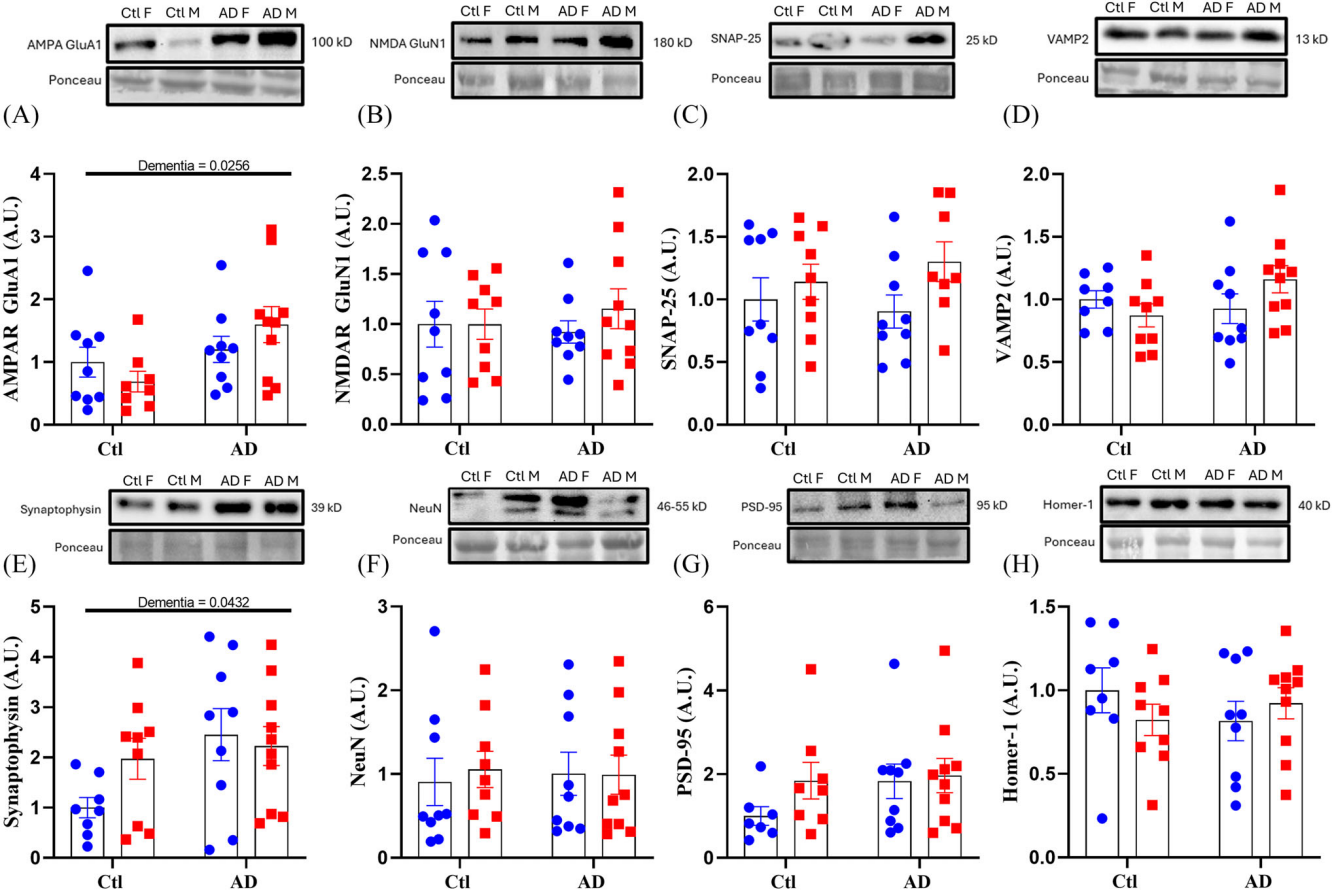
Select synaptic markers in the PFC have greater expression with AD. Changes in brain synaptic markers for human PFC with and without AD: (A) total SNAP‐25 protein content, (B) total VAMP2 protein content, (C) total AMPAR GluA1 protein content, (D) total NMDAR GluN1 protein content, (E) total PSD‐95 protein content, (F) total Homer‐1 protein content, (G) total NeuN protein content, and (H) total synaptophysin protein content. Proportional change (%) represented as mean ± standard error of the mean. Differences between groups as determined using a two‐way analysis of variance followed by Fisher least significant difference post hoc analysis. AD, Alzheimer's disease; AMPAR, α‐amino‐3‐hydroxy‐5‐methyl‐4‐isoxazolepropionic acid receptor; Ctl, control; NMDAR, N‐methyl‐D‐aspartate receptor; PFC, prefrontal cortex; SNAP‐25, synaptosomal‐associated protein of 25 kDa; VAMP2, vesicle‐associated membrane protein 2.

With respect to synaptic and neuronal protein content, there were main effects for dementia status for two proteins: α‐amino‐3‐hydroxy‐5‐methyl‐4‐isoxazolepropionic acid (AMPA)‐receptor GluA1 (a key glutamatergic synapse marker; dementia, *F*[1, 32] = 5.480, *p* = 0.0256) and synaptophysin (a general synaptic marker; dementia, *F*[1, 32] = 4.433, *p* = 0.0432). Both proteins had higher expression for both AD male and female individuals compared to controls (Figure 3A, E). No other synaptic or neuronal markers tested were found to be different between groups (Figure 3B, C, D, F, G, and H) indicating that contrary to previous reporting,^45^ AD does not appear to reduce the expression of key neuronal and synaptic proteins and in some instances results in an increase in expression.

**FIGURE 4:**
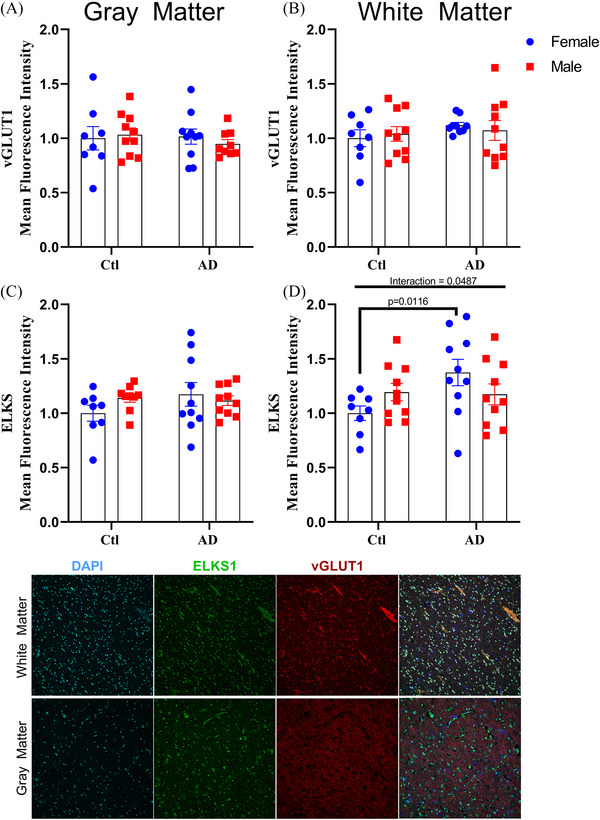
Females with AD had greater ELKS expression in the white matter of the PFC. Total mean fluorescence intensity for (A,B) vGLUT1 and (C,D) ELKS. DAPI shown in blue, ELKS1 shown in green, vGLUT1 shown in red; *n* = 38 per region. Percent change represented as mean ± standard error of the mean. Differences between groups as determined using a two‐way analysis of variance followed by Fisher least significant difference post hoc analysis. AD, Alzheimer's disease; Ctl, control; DAPI, 4',6‐diamidino‐2‐phenylindole; PFC, prefrontal cortex; vGLUT1, vesicular glutamate transporter 1.

As western blotting relies on the homogenization and analysis of the PFC, there is a possibility that regional specific differences in synaptic proteins might be obscured. As such, immunofluorescence analysis of vGLUT1 (required for synaptic‐vesicle glutamate shuttling) and ELKS1 (required for shuttling synaptic vesicles to synapses) was conducted between the gray and white matter of the PFC. Total mean fluorescence intensity of vGLUT1 and ELKS1 revealed that within gray matter there was no difference in expression of either vGLUT1 or ELKS1 (Figure 4A and B), but within white matter ELKS1 showed a significant two‐way interaction (Figure 4B and D; interaction, *F*[1, 34] = 4.182, *p* = 0.0487), in which AD females showed higher ELKS1 expression compared to control females (Figure 4D; *p* = 0.0116). This increase in ELKS1 mirrors the increases in both AMPA and synaptophysin content that was seen for both males and females with AD, highlighting that some synaptic proteins exhibit a greater expression with AD despite the known impact of AD on synaptic and neuronal survival.^43^, ^45^ However, the increase in ELKS1 expression only being found in the white matter of AD females indicates that there may be a sex‐ and region‐specific response toward AD within the brain.

### 3.4 Microglia and inflammatory cytokines

**FIGURE 5:**
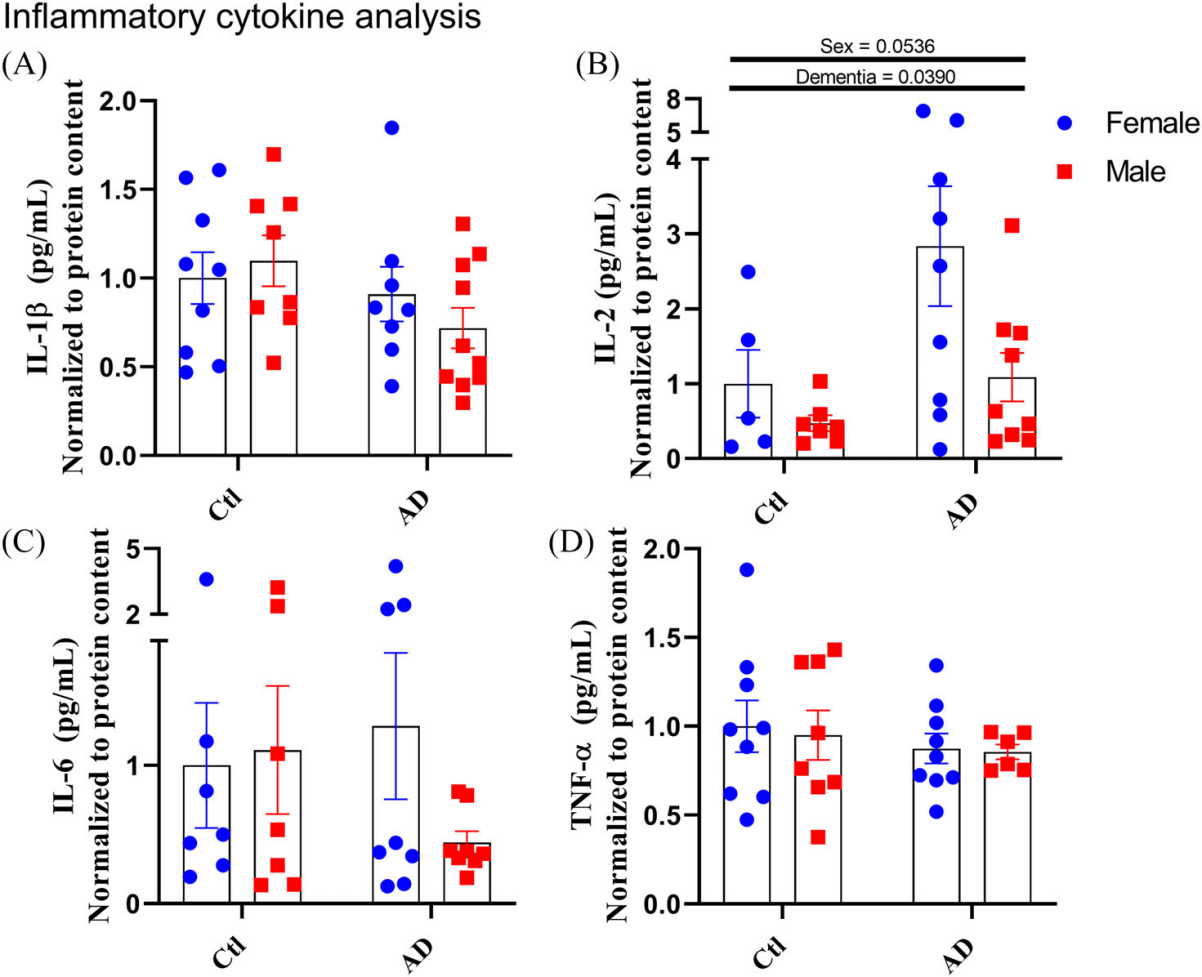
Sex and dementia‐induced changes in IL‐2, but not TNF‐α. Inflammatory cytokine panel analysis for PFC homogenate: (A) IL‐1β, (B) IL‐2, (C) IL‐6, (D) TNF‐α. Normalized mediator concentration represented as mean ± standard error of the mean. Differences between groups as determined using a two‐way analysis of variance followed by Fisher least significant difference post hoc analysis. AD, Alzheimer's disease; Ctl, control; IL, interleukin; PFC, prefrontal cortex; TNF‐α, tumor necrosis factor alpha.

Neuroinflammation has been identified as a pathology that has significant contributions to AD progression, particularly from Aβ peptide–driven pro‐inflammatory cytokine secretion and the activation of microglia which have been linked to synaptic degradation and neuronal death.^2^ However, changes in these markers have rarely been reported in humans,^46^ and have not, to our knowledge, been examined by sex. As such, we explored whether males and females exhibit differences in the pro‐ and anti‐inflammatory cytokine content within the PFC as well as differences in Iba1 expression (a marker of microglial content and inflammatory activation^32^, ^35^). The levels of human cytokines IL‐1β, TNF‐α, or IL‐6 showed no differences between groups (Figure 5A, C, D). However, IL‐2, a potent activator of T‐cell differentiation and proliferation,^47^ showed a significant main effect for dementia status (Figure 5B; dementia, *F*[1, 26] = 4.728, *p* = 0.0390), whereby AD individuals had higher IL‐2 content compared to controls. There was a trending main effect for sex (Figure 5B; sex, *F*[1, 26] = 4.088, *p* = 0.0536) for which females showed higher IL‐2 content compared to males. Overall, the cytokine analysis indicates that contrary to previous reporting in animal models,^48^ humans with AD do not exhibit an increase in pro‐inflammatory cytokines compared to non‐AD individuals but in fact show higher IL‐2, which may also be greater in AD females.

**FIGURE 6:**
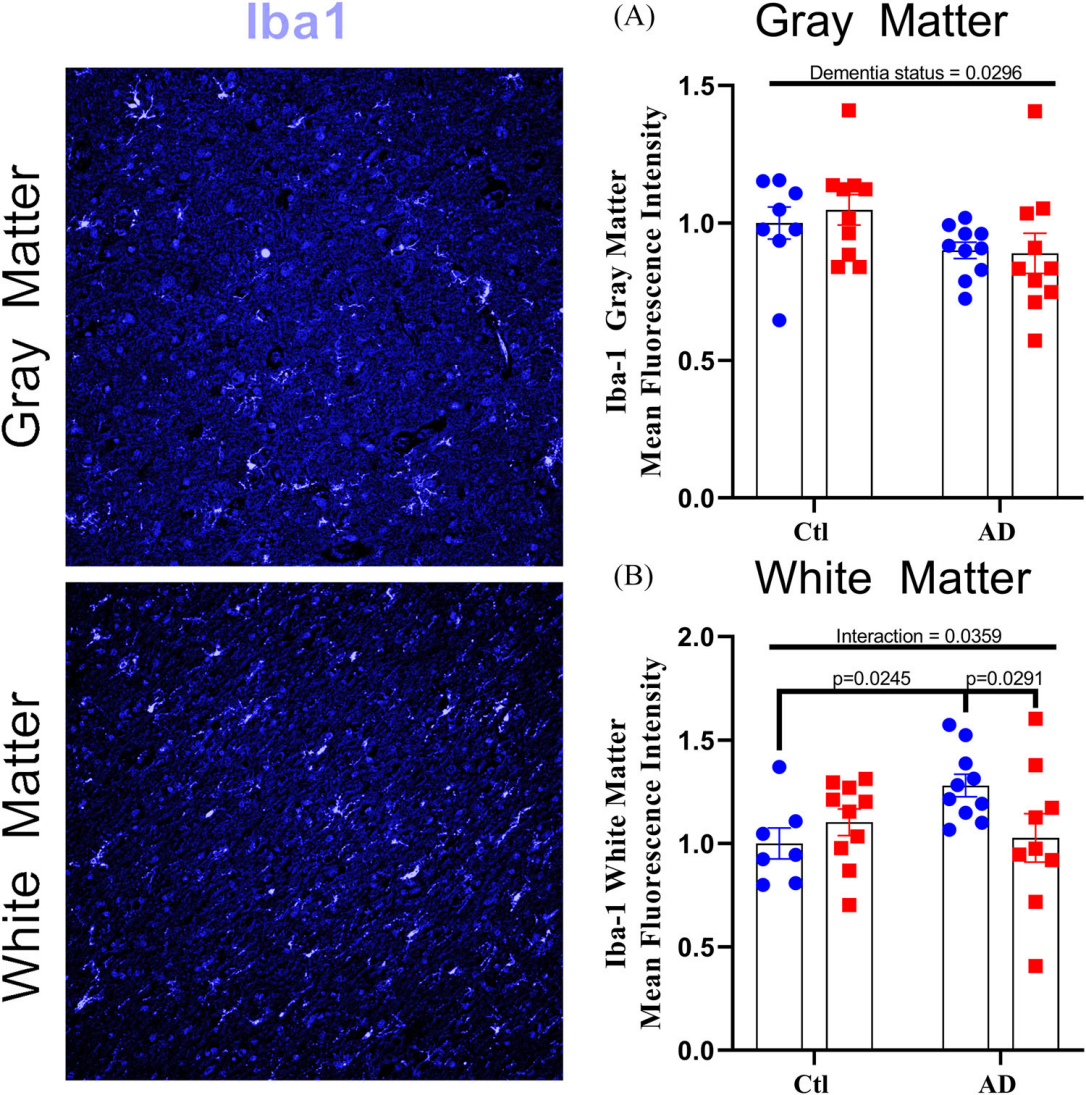
Females have greater Iba1 expression in white matter with AD. Total mean fluorescence intensity for Iba1 within (A) white and (B) gray matter; *n* = 38 per region. Percent change represented as mean ± standard error of the mean. Differences between groups as determined using a two‐way analysis of variance followed by Fisher least significant difference post hoc analysis. AD, Alzheimer's disease; Ctl, control; Iba1, ionized calcium‐binding adaptor molecule 1.

Total mean fluorescence intensity for Iba1 showed a main effect for dementia in gray matter (Figure 6A; dementia, *F*[1, 34] = 5.156, *p* = 0.0296), with a lower expression of Iba1 in AD individuals suggesting a reduction in the number of “activated” microglia in the gray matter of AD individuals. In white matter, there was a significant two‐way interaction between Iba1 (Figure 6B; interaction, *F*[1, 32]) = 4.795, *p* = 0.0359), whereby AD females showed higher Iba1 expression than both control females (Figure 6B; *p*_Iba1 _= 0.0245) and AD males (Figure 6B; *p*_Iba1 _= 0.0291). This greater expression of Iba1 in the white matter of AD females indicates that AD impacts microglial expression in females differently than men (possibly increasing their inflammatory activity), but only in the white matter.

**FIGURE 7:**
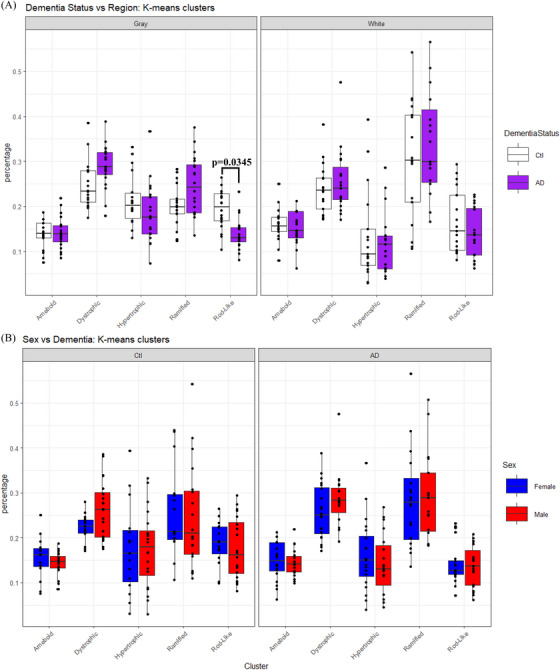
Rod‐like microglial population is reduced with AD. Analysis of microglial morphological changes in the PFC with and without AD. Results are separated by (A) dementia status (white = control, purple = AD) and (B) separated by sex (blue = female, red = male). Microglial analysis was conducting using the MicrogliaMorphology R package designed by Kim et al.^32^ Each *N* represents technical replicates of between ≈ 1000 to 2000 individual microglia per morphological cluster per group. Percent change represented as mean ± standard error of the mean. Differences between groups as determined using a three‐way analysis of variance followed by Bonferroni post hoc analysis. AD, Alzheimer's disease; Ctl, control; PFC, prefrontal cortex.

Analysis of Iba1 expression cannot be used to describe the extent of microglial activation within the brain; microglial function, either pro‐ or anti‐inflammatory, is more highly correlated to morphology than the expression of Iba1.^32^, ^35^ Therefore, we also examined changes in microglial morphology between male and female AD individuals in both the gray and white matter of the PFC.^32^ When analyzing microglial morphology by dementia status versus region, there was a significant two‐way interaction between cluster and dementia status (cluster vs. dementia, *F*[4, 334] = 16.4202, *p* = 0.0025) for which a significant difference in the number of rod‐like microglia was found: AD individuals showed fewer rod‐like microglia compared to controls within gray matter (Figure 7A; *p* = 0.0345). No differences in morphology were observed in white matter. Additional analysis of microglial morphology by sex versus dementia demonstrated that there were no differences in morphology by sex (Figure 7B, cluster vs. sex, *F*[4, 334] = 4.4229, *p* = 0.3517). Analysis of microglial morphology indicates that, while there are significant differences in Iba1 content for both gray and white matter, these changes do not translate to differences in microglial morphology for AD males and females. The reduction in rod‐like microglia that are contrary to previous reporting,^49^ alongside a lack of changes in any other microglial morphology with AD, indicate that AD does not significantly modify the population of microglial morphologies in humans.

## 4 DISCUSSION

This work identified differences in human *post mortem* AD brains related to insulin signaling, mitochondrial function and protein expression, synaptic protein expression, and markers of neuroinflammation. Specifically, in the PFC of humans with AD, we demonstrate greater Akt S473 phosphorylation, greater content of OXPHOS complexes III and V paired with lower OCR through complex I and III, greater AMPA GluA1 and synaptophysin expression, greater anti‐inflammatory IL‐2 content, and lower expression of Iba1 in the gray matter of human AD individuals. Further, females with AD were found to exhibit unique changes in metabolic markers that differed significantly from males, suggestive of sex‐specific differences in metabolic capacity.

Besides Akt S473 phosphorylation,^10^, ^11^ the majority of these results contradict previous work that reports reductions in mitochondrial OXPHOS complex content (complex III and V protein content),^8^, ^50^ reductions in neuronal/synaptic markers,^45^, ^51^, ^52^ increases in pro‐inflammatory cytokine expression,^48^, ^53^ and greater microglial activation^2^, ^54^ in induced pluripotent stem cell, rodent, and *post mortem* human brains. Additionally, some increases could be described as beneficial: greater expression of complex III and complex V could suggest that AD individuals have a greater OXPHOS capacity to improve ATP synthesis^41^, ^42^ and greater AMPA GluA1 and synaptophysin expression could suggest a greater synaptic organization that favors improved transmission.^55^ However, as we observed impaired OCR capacity through complex I and complex III in AD brains, we suggest that the increases in OXPHOS complex expression could be compensatory increases to maintain mitochondrial function when presented with metabolic impairment. Similarly, increases in AMPA and synaptophysin content might describe a consolidation of synaptic machinery to maintain existing synaptic function,^56^, ^57^, ^58^, ^59^, ^60^ possibly indicative of a compensatory response to loss of neurons or synapses. However, AMPA receptors are also located in the extra‐synaptic regions of neurons where they drive excitotoxicity.^61^ Excitotoxicity is a prominent feature of AD pathology^43^ and might suggest that the increases in GluA1 that we and others^61^ identify are a pathological response to AD development or contribute to neuronal loss. Similarly, given the role of synaptophysin in the endocytosis of secreted synaptic vesicles, increases in synaptophysin might also describe an attempt by neurons to increase the re‐uptake of synaptic vesicles in the response to rampant synaptic vesicle release that is known to be associated with Aβ production.^43^

AD brains have been shown to have an upregulation of pro‐inflammatory cytokines such as IL‐6, IL‐1β, and TNF‐α.^48^, ^53^ However, only increases in the cytokine IL‐2 were observed in the present study. IL‐2 is a major cytokine responsible for driving inflammatory signaling through the differentiation of CD4+ T helper cells as well as influencing the differentiation/proliferation of memory and effector CD8+ T cells.^62^ Because of this role, IL‐2 is often associated with a powerful pro‐inflammatory immune response. However, as a result of complex interactions between IL‐2 and T cells, varying concentrations of IL‐2 have been shown to regulate both pro‐inflammatory or anti‐inflammatory actions.^63^ Within the context of the AD brain, low‐dose IL‐2 secretion has been described to downregulate pro‐inflammatory signaling,^64^ prevent prolonged pro‐inflammatory damage,^47^ prevent synaptic failure, and improve memory in mice.^65^ As such, an overexpression of IL‐2 might occur to regulate an increased neuroinflammatory environment driven by AD progression. This idea is supported by a higher baseline expression of IL‐2 being associated with less cognitive decline over time in humans,^66^ improvements to cognitive function after adeno‐associated virus IL‐2 expression in mice,^47^ and the importance of IL‐2 in regulating Treg cell populations to repress overactive glial responses to brain insults such as AD.^67^ Despite this, a more detailed examination is required to confirm whether increases in IL‐2 observed in this study reflect similar outcomes described in previous studies. Specifically, analysis of the impact that IL‐2 has on astrocytes is of interest as astrocytic activation by IL‐2 has been linked to AD improvements.^47^

Chronic microglial activation has also been associated with mitochondrial^2^, ^9^, ^15^ and synaptic damage.^2^, ^17^, ^18^ Variations in microglial morphology have also been identified in AD cases,^36^ though rarely reported in humans.^14^ We found lower Iba1 content in the gray matter of AD patients and higher Iba1 in the white matter in males with AD. These differences in Iba1 expression between the gray and white matter indicate that microglial activation may be region specific and point to differences in inflammatory environments. Given the importance of microglia in regulating the inflammatory response of the brain,^35^ lower content of Iba1 in the gray matter of AD individuals could imply a reduction in the activation of microglia with AD.^68^ However, while literature has reported increases in Iba1 within human AD brains, significant reductions have also been shown.^69^ Additionally, the observation for lower rod‐like microglia in AD gray matter, while conflicting with previous reports in humans,^49^ supports the idea of region‐specific microglial responsiveness.

We observed distinct sex‐specific differences between males and females both with and without AD. Although dysregulated insulin signaling has been linked to AD progression^11^ with respect to sex, these findings have not been explored. We found that males had dysregulated metabolic signaling, where AD males show the expected greater phosphorylation in mTOR S2448.^11^ However, this increase was not seen with AD females, despite both AD groups showing greater upstream Akt S473 phosphorylation. This suggests that with AD, the female brain does not exhibit the same response to changes in Akt signaling as males. Other metabolic differences found point to AD females possessing an impaired and unique brain metabolism compared to AD males. While sex‐specific reductions in OCR through complex IV were found with AD, when normalized to the protein expression of the complexes these differences were absent. Instead, differences in complex III respiration were seen, seemingly driven by sex, control female OCR was significantly higher than all other groups and exhibited the largest reduction in OCR with AD while no difference in complex III respiration was observed between control or AD males. The results of both the normalized and non‐normalized respiration point to a sex‐specific difference in the brain metabolism of females with AD for which female brains are more sensitive to metabolic impairments within AD—an idea that is supported by the possible higher anti‐inflammatory IL‐2 cytokine expression in female brains. As such, the higher expression of IL‐2 within females, exacerbated with AD, could suggest a greater sensitivity of females toward changes in the inflammatory environment.

Similarly, the sex‐specific differences in ELKS1 protein expression might represent AD‐specific compensatory signaling within the PFC. ELKS1 is a presynaptic protein that regulates the population of readily available synaptic vesicles at excitatory synapses.^70^, ^71^ The higher ELKS1 content seen in female AD brains in this study could suggest an increased synaptic vesicle regulation that would prime synapses for greater vesicle release.^70^, ^71^ However, as the AD individuals in this study presented with cognitive impairment, this increase in ELKS1 content is unlikely to represent improved brain function. Instead, given its role is synaptic transmission, this higher ELKS1 content likely describes a compensatory increase in a key presynaptic protein in an effort to stabilize synaptic function in the face of ongoing damage/cognitive decline in a similar mechanism as described by Goel et al.^72^ However, as these protein changes were not correlated with any functional data it is unclear whether these increases in protein expression indicate a compensatory increase in synaptic machinery.^72^

Additionally, similar justification could be suggested for the higher expression of Iba1 in the white matter of AD females. Damaging pro‐inflammatory microglial activation has been reported in multiple dementias,^32^, ^73^ which alongside the importance of microglial pruning of synapses and the greater proportion of axonal tracts that exist in white matter, could describe a sex‐specific increase in microglial pruning^2^, ^18^ in response to AD progression. This would be in line with increased levels of the cytokine IL‐2, as well as a justification for the greater white matter ELKS1 expression of AD females only: greater anti‐inflammatory cytokine production and pre‐synaptic ELKS1 expression are a compensatory response to maintain or improve the damage in the white matter driven by microglial activation. Additionally, the differences in both synaptic and inflammatory responses, when coupled with the differences in mitochondrial function, highlight a sex‐specific difference in the brain environments between males and females in which female brain metabolism is particularly impacted by AD progression.

A limitation with working with *post mortem* human brains is that there may be differences in protein expression due to factors such as age, medical history, and the time between death and tissue preservation or PMD (Table S1 in Supporting Information). Despite efforts to control variables, some variability in protein expression remains in representative western blots, reflecting a common challenge when working with *post mortem* tissue. While there may be a reduction in protein expression due to PMD as described previously,^74^, ^75^, ^76^ this reduction in protein expression would be expected to be similar across all samples, thus not affecting our overall conclusions. Additionally, microglial morphology may be altered by PMD; however, similar to the results related to protein content alterations in morphology would be expected to be similar across all samples.

This study found that AD brains have distinct metabolic and neuroinflammatory environments compared to controls wherein AD brains present with worse metabolic dysregulation and greater neuroinflammation. Importantly, we also provide evidence that female AD brains are more metabolically dysregulated than males but that female brains may also possess a greater compensatory response to AD progression that likely occurs through a separate mechanism from males. These differences in brain environments highlight the importance of metabolic health in the brain and describe how improvements to the metabolic environment of the brain might represent a promising target for AD therapy, particularly among females.

## CONFLICT OF INTEREST STATEMENT

The authors have no conflicts of interest or disclosures to declare. Author disclosures are available in the Supporting Information.

## Supporting information



Supporting Information

Supporting Information

Supporting Information

Supporting Information

Supporting Information
