# Supplementary material for: Differences in inflammatory markers, mitochondrial function, and synaptic proteins in male and female Alzheimer's disease post mortem brains
Source: Alzheimers Dement. 2025 Oct 1;21(10):e70645. doi: 10.1002/alz.70645 (PMC12485286; doi:10.1002/alz.70645)
Supplement: Supplementary file 1 — Supporting Information [file ALZ-21-e70645-s003.pdf]

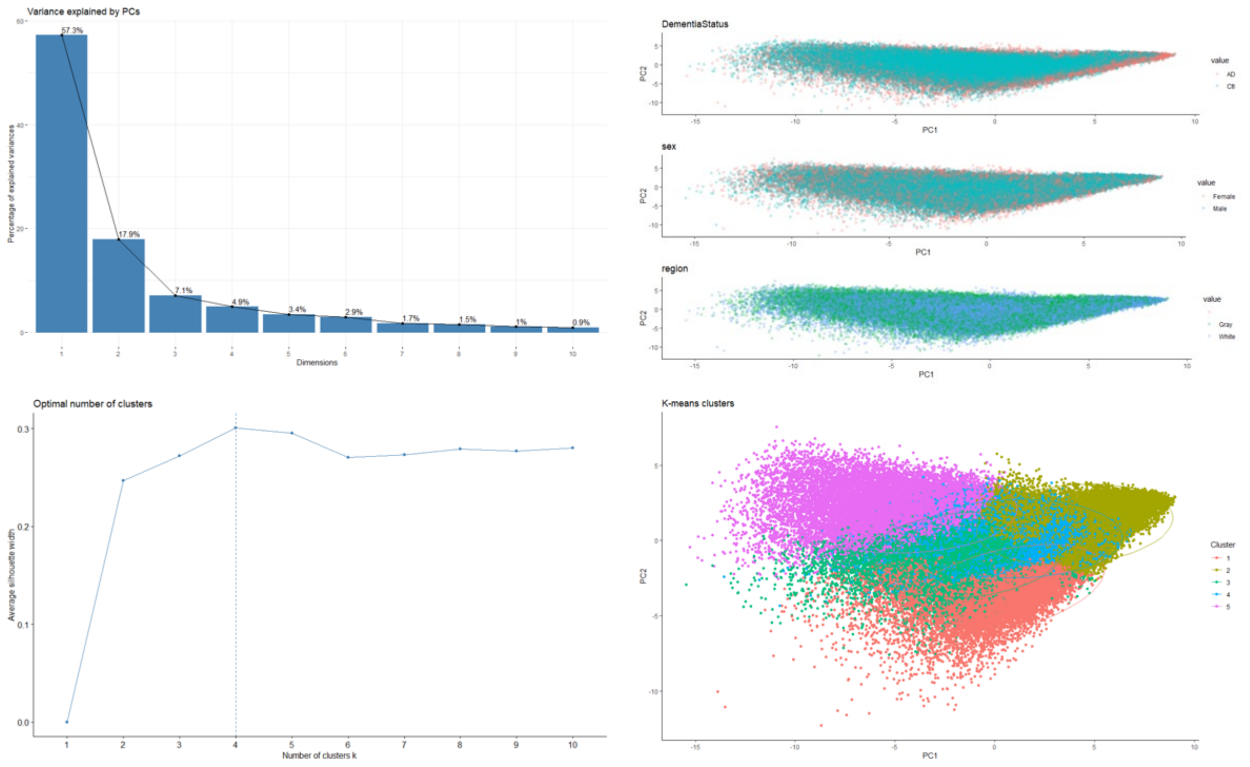

Supplemental Figure 1: Percent variability by cluster and sources of variability in data set. A: The amount of variability within the data set described by principal component analysis; the optimal number of PCs required for downstream analysis encompasses the majority of variability in the data. B: Visual representation of the percent variability (PC1) within the data set by independent variables (age, exercise, region). C: K-means clustering based on PCs; number of optimal clusters (K) needed to describe the variability within the data set within the optimal number of PCs described in A. D: Visual representation of the percent variability (PC1) within the data set by the optimal number of clusters described in C (5).
