# Supplementary material for: Differences in inflammatory markers, mitochondrial function, and synaptic proteins in male and female Alzheimer's disease post mortem brains
Source: Alzheimers Dement. 2025 Oct 1;21(10):e70645. doi: 10.1002/alz.70645 (PMC12485286; doi:10.1002/alz.70645)
Supplement: Supplementary file 2 — Supporting Information [file ALZ-21-e70645-s004.pdf]

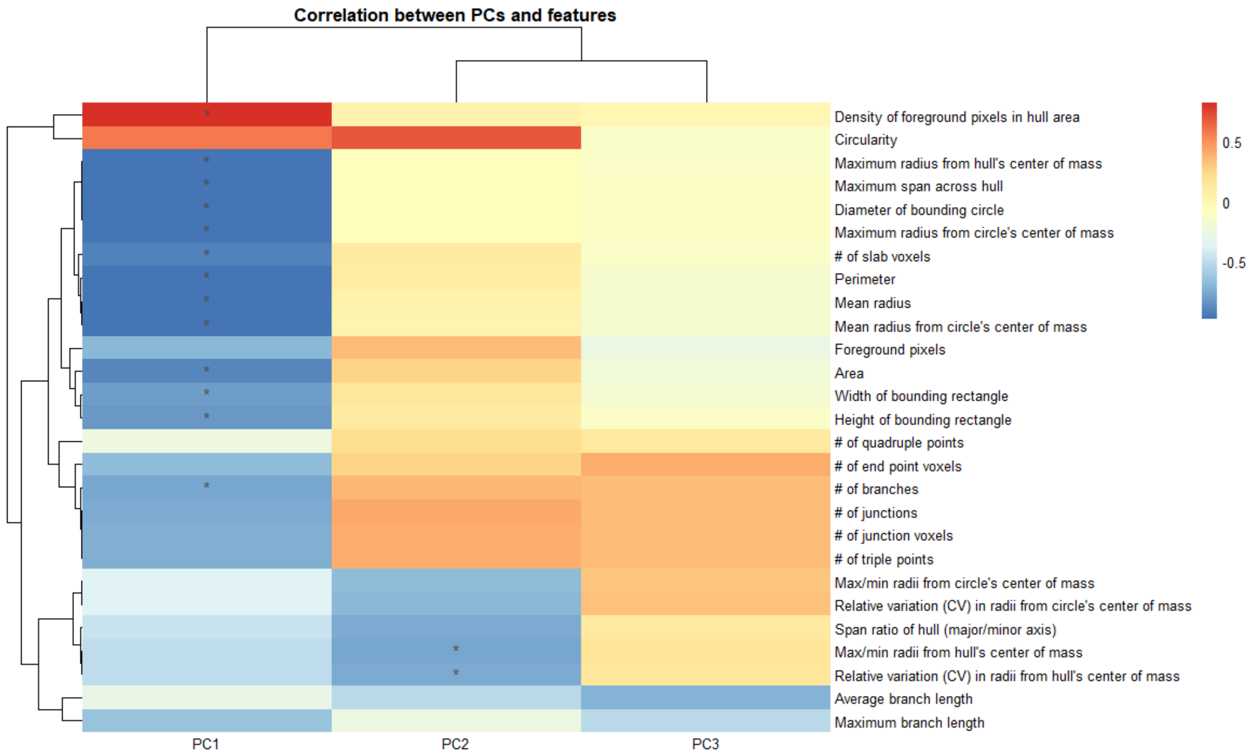


Supplemental Figure 2: Heatmap of correlations between principal component clusters and 27 individual microglial morphological measures. PC1 variability described by highly negatively correlated to features for branching complexity and territory span, meaning that individual cells with greater branching complexity or area have lower PC1 scores in our dataset. PC2 variability described by cell shape: 1) *circularity* (circularity, max/min radii from center, span ratio of hull) and 2) *branching homogeneity* (relative variation (CV) from center of mass), and PC3 is described by branch length-related measures.
