# Supplementary material for: Differences in inflammatory markers, mitochondrial function, and synaptic proteins in male and female Alzheimer's disease post mortem brains
Source: Alzheimers Dement. 2025 Oct 1;21(10):e70645. doi: 10.1002/alz.70645 (PMC12485286; doi:10.1002/alz.70645)
Supplement: Supplementary file 3 — Supporting Information [file ALZ-21-e70645-s001.pdf]

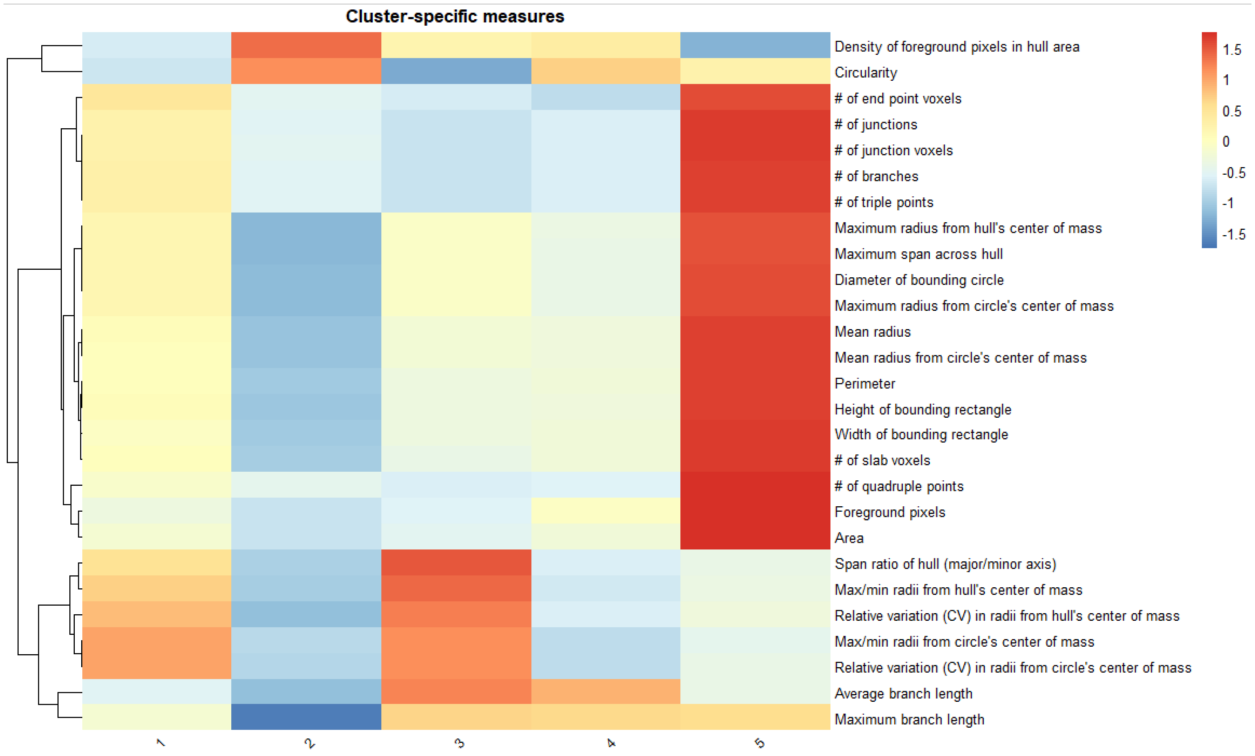

Supplemental 3: Percent cluster analysis describing % variability of interest for defining major morphological states of microglia derived from 27 individual morphological markers. PC1 = Dystrophic (lowest branch thickness and length as explained by pixel density in hull), PC2 = ameboid (lowest territory span, high circularity, smallest branch lengths), PC3 = rod-like (greatest oblongness, lowest circularity), PC4 = Hypertrophic (average territory span, high branch thickness as explained by pixel density in hull), small cell bodies, large number of branches and junctions) PC5 = ramified (largest territory span and branching complexity)
