# Supplementary material for: Differences in inflammatory markers, mitochondrial function, and synaptic proteins in male and female Alzheimer's disease post mortem brains
Source: Alzheimers Dement. 2025 Oct 1;21(10):e70645. doi: 10.1002/alz.70645 (PMC12485286; doi:10.1002/alz.70645)
Supplement: Supplementary file 4 — Supporting Information [file ALZ-21-e70645-s005.docx]

**Supplementary Table 1** Case information on samples used in the study. Samples and their neuropathological reports were provided by The Douglas-Bell Canada Brain Bank and assessed by a board-certified pathologist.

| **Case** | **Pathological Diagnosis** | **Sex** | **Age** | **PMD (Hours)** | **Neuropathological results** |
| --- | --- | --- | --- | --- | --- |
| 1 | Control | F | 80 | 17.5 | Cerebral senile changes, non-specific, mild to moderate, very predominantly limbic; Absence of specific pathological changes, with normal associative cortex. |
| 2 |  |  | 71 | 10.83 | Mild, acute ischemic anoxic encephalopathy; very mild, non-specific senile brain changes. |
| 3 |  |  | 72 | 13.42 | Acute ischemic anoxic encephalopathy; Senile, nonspecific, minimal cerebral changes; Arteriosclerosis of small cerebral arteries, suggestive of chronic arterial hypertension with mild perivascular white matter gliosis. |
| 4 |  |  | 71 | 13.5 | Mild to moderate acute ischemic anoxic encephalopathy; Mild nonspecific senile cerebral changes, mainly limbic; Isolated old lacunar infarct, left caudate nucleus; Mild atherosclerosis of small cerebral arteries. |
| 5 |  |  | 75 | 6.75 | Moderate to severe, nonspecific senile cerebral changes, targeted mainly on the amygdala, Ammon's horn and parasubiculum; Mild, senile amyloid angiopathy, mainly leptomeningeal; Arterosclerosis of small cerebral arteries, with perivascular gliosis of white matter, is generally moderate |
| 6 |  |  | 92 | 16.5 | Brain showing plaques and tangles consistent in quantity with the age of the patient; Congophilic angiopathy minimal; Atrophy of superior vermis minimal. |
| 7 |  |  | 78 | 8 | Acute, generalized, severe ischemic anoxic encephalopathy; Personal history of multiple severe depressions without evidence of cognitive deficit, with : A) Multifocal cortical neuronal loss associated with mild reactive gliosis. B) Absence of specific histological and/or immunohistochemical lesions. C) Lesions predominantly in fronto-temporo-parietal associative cortex; Neurodegenerative lesions of normal aging predominantly limbic, mild, non-specific; Atrophy of superior vermis, severe, non-specific. |
| 8 |  |  | 85 | 12 | Anoxic-ischemic encephalopathy, acute, moderately severe; Non specific neurodegenerative lesions, predominantly limbic, consistent with normal aging; Artériosclerosis of large and small cerebral arteries, mild to moderate. |
| 9 |  |  | 79 | 17.3 | Age-related neurodegenerative changes; very discreet hyaline arteriolosclerosis. |
| 10 |  |  | 91 | 17.75 | Cerebral atrophy, frontal, temporal and parietal, moderate to marked; Cerebrovascular atherosclerosis, moderate; Congophilic angiopathy, moderate |
| 11 |  | M | 69 | 12 | Cerebral senile changes, non-specific, mild; Anoxic ischemic encephalopathy, acute, mild to moderate; Senile amyloid angiopathy, very focal and mild |
| 12 |  |  | 88 | 15.98 | Mild, nonspecific senile brain changes; Mild ischemic anoxic encephalopathy. |
| 13 |  |  | 76 | 20.5 | Acute, generalized, severe ischemic anoxic encephalopathy; Multiple ancient lacunar infarcti, predominant in basal ganglia, especially striatum, multiple; Ancient cortical infarcti, predominantly frontal and parietal, microscopic, multiple. A) Focal, substantia nigra; Arterial and arteriolar sclerohyalinosis, severe, compatible with chronic arterial hypertension; Non-specific degenerative lesions of aging, predominant in limbic lobes, mild. |
| 14 |  |  | 81 | 21.5 | Marked vascular sclerohyalinosis with multifocal microscopic ancient cortical infarcts and microscopic ancient lacunar infarcts of the basal ganglia; Absence of neurodegenerative changes; Terminal acute anoxic-ischemic encephalopathy. |
| 15 |  |  | 80 | 13 | Cerebral vascular atherosclerosis, moderate; Brain showing no evidence of dementing disease. |
| 16 |  |  | 93 | 18 | Ancient cavitary infarction extending over the territory of the left middle cerebral artery; Ancient infarction of the left cerebellar cortex; Marked arteriolosclerosis of small intracerebral vessels; Presence of small, microscopic, multifocal ancient infarcts of the basal ganglia; Absence of neurodegenerative changes. |
| 17 |  |  | 83 | 16.83 | Absence of neurodegenerative changes; Amyloid angiopathy; Histological changes suggestive of metabolic encephalopathy; Terminal acute anoxic-ischemic encephalopathy. |
| 18 |  |  | 72 | 7.48 | Histological changes suggestive of metabolic encephalopathy; End-stage acute anoxic-ischemic encephalopathy; Absence of neurodegenerative changes; Absence of metastatic neoplasia |
| 19 |  |  | 76 | 19.75 | Hypoxic-anoxic encephalopathy: Severe vacuolization and neurons with hypereosinophilic, wilted cytoplasms found at supra- and infratentorial levels; Cerebrovascular pathology: Hypertensive and/or atherosclerotic and/or diabetic vasculopathy; Absence of associated proteinopathy (Tau, beta-amyloid, TDP-43, alpha-synuclein). |
| 20 |  |  | 68 | 23.37 | Atherosclerotic and/or chronic hypertensive and/or diabetic vasculopathy associated with decreased myelin content in telencephalic white matter; Absence of clinically significant Alzheimer-type neuropathological changes; Absence of associated hippocampal sclerosis; Absence of Lewy body disease (a-synucleinopathy); Absence of associated TDP-43 proteinopathy. |
| 21 | AD | F | 80 | 15 | Plaque-predominant senile Alzheimer's disease with: a) Moderate atrophy and reactive gliosis of the entorhinal cortical ribbon and frontotemporal associative isocortex; b) Presence of moderate amounts of senile plaques in the same regions; Moderate arteriolosclerosis and atherosclerosis of small intracerebral vessels; Acute terminal ischemic anoxic encephalopathy. |
| 22 |  |  | 81 | 20.5 | High-grade Alzheimer's disease according to the ABC classification (A3, B3, C3); Amyloid angiopathy; Moderate to marked arteriolosclerosis with old cortical microscopic infarcts of the associative isocortex and cerebellar cortex that may have contributed to cognitive impairment. |
| 23 |  |  | 78 | 19.42 | High-risk Alzheimer's disease changes according to ABC classification (A3, B3, C3); Amyloid angiopathy; Moderate to marked arteriolosclerosis of small intracerebral vessels; Presence of old multifocal intracortical microscopic infarcts; Old infarcts of cerebellar cortex. |
| 24 |  |  | 79 | 18.17 | High-risk Alzheimer's disease according to ABC classification (A3, B3,.C3); Amyloid angiopathy; Moderate arteriolosclerosis of small intracerebral vessels; Acute terminal anoxic-ischemic encephalopathy. |
| 25 |  |  | 93 | 10.35 | "Alzheimer's" type neuropathological changes associated with a "high" probability of clinical dementia: Thal Stage 4 (A3), Braak and Braak Stage V/VI (B3), Age-weighted CERAD Score C (C3): A3, B3, C3; Cerebrovascular pathology: - Atherosclerotic and/or hypertensive vasculopathy; Absence of associated TDP-43 proteinopathy; Absence of associated Lewy body disease (alpha-synucleinopathy); Absence of associated hippocampal sclerosis |
| 26 |  |  | 74 | 8.8 | Alzheimer's-like neuropathological changes associated with a high probability of clinical dementia: A3, B3, C3; Associated hypertensive and/or atherosclerotic and/or diabetic vasculopathy; Absence of associated Lewy body disease (alpha-synucleinopathy); Absence of associated hippocampal sclerosis. |
| 27 |  |  | 75 | 7.5 | Neurodegenerative changes of high risk for Alzheimer's disease according to the ABC classification (A3, B3, C2); Amyloid angiopathy; Moderate arteriolosclerosis of small intracerebral vessels. |
| 28 |  |  | 86 | 10.54 | Neuropathological alterations of Alzheimer's disease (A3 B3 C3: Alzheimer's disease) |
| 29 |  |  | 92 | 14.38 | Senile Alzheimer's disease: Atrophy and moderate gliosis of the entorhinal cortical ribbon and the frontotemporal associative isocortex. Presence of senile plaques and neurofibrillary tangles in marked quantity in the same regions; Amyloid angiopathy; Marked arteriolosclerosis of the small intracerebral vessels; Mild chronic ischemic leukoencephalopathy. |
| 30 |  |  | 74 | 22.28 | "Alzheimer's" type neuropathological changes associated with a "high" probability of clinical dementia: A3, B3, C3; Cerebrovascular pathology: Atherosclerotic and/or chronic hypertensive and/or severe diabetic vasculopathy; Absence of associated TDP-43 proteinopathy; Absence of associated Lewy body disease (alpha-synucleinopathy); Absence of associated hippocampal sclerosis. |
| 31 |  | M | 79 | 12.25 | Diffuse and marked Tauopathie associated with diffuse hippocampal and neocortical amyloid deposits. No amyloid angiopathy is seen. These neuropathological changes are in favor of an Alzheimer disease with advanced pathologic changes (severe Alzheimer disease). ABC final Score: A2 B3 C2; Willis circle examination reveals the presence of partially calcified atheromatous plaques with narrowing of the arterial lumen below 25%. No aneurism dilatation is noted; No evidence of cerebrospinal fluid accumulation or blocking of the cerebrospinal circulation is noted; No hypoxic or ischemic lesions are seen; No neoplastic lesion is noted. |
| 32 |  |  | 87 | 10.8 | Senile Alzheimer's disease with a) Atrophy and marked reactive gliosis of the entorhinal cortical ribbon and frontotemporal associative isocortex; b) Presence of moderate to marked senile plaques and neurofibrillary degeneration in the same regions; Amyloid angiopathy; Moderate to marked arteriolosclerosis and atherosclerosis of small intracerebral vessels; Subacute hemorrhagic microscopic infarction of the right cerebral peduncle and ventral substantia nigra; Acute terminal ischemic anoxic encephalopathy. |
| 33 |  |  | 87 | 15.17 | Cerebrovascular disease, with: Atherosclerosis of internal carotid artery and arteriolosclerosis, severe, in cerebral white matter and basal ganglion; Alzheimer's disease, neuropathological ''intermediate'' changes : A2, B3, C3, with cerebral amyloid angiopathy. |
| 34 |  |  | 89 | 22.83 | High-risk neurodegenerative changes according to ABC classification (A3, B3, C3) **Alzheimer's disease mentioned in comments*; Microvascular changes with early multifocal microscopic infarcts of cortical ribbon and hippocampus testifying to microvascular pathology having contributed to cognitive impairment; Marked arteriolosclerosis of small intracerebral vessels |
| 35 |  |  | 75 | 20.87 | Alzheimer's disease neuropathological changes (High) : A3, B3, C3, with : cerebral amyloid angiopathy |
| 36 |  |  | 80 | 18.83 | Neuropathological alterations of Alzheimer's disease (A3 B3 C3: Alzheimer's disease, high probability); Focal hyaline arteriolosclerosis |
| 37 |  |  | 87 | 13.72 | Senile Alzheimer's disease (A3, B3, C3; High probability); Amyloid angiopathy; Moderate to marked arteriolosclerosis of small intracerebral vessels; Old lacunar infarct of the right anterior caudate nucleus. |
| 38 |  |  | 88 | 18.13 | Neuropathological alterations of Alzheimer's disease (A3 B3 C2); Diffuse, multifocal hyaline arteriolosclerosis. A) Diffuse rarefaction of white matter |
| 39 |  |  | 73 | 16.42 | 'Alzheimer's'-like neuropathological changes associated with a 'high' probability of clinical clemency: A3, B3, C3; Cerebrovascular pathology: Atherosclerotic and/or chronic hypertensive and/or diabetic vasculopathy; Absence of associated hippocampal sclerosis; Absence of Lewy body disease (α-synucleinopathy); Absence of associated TDP-43 proteinopathy. |
| 40 |  |  | 79 | 11.86 | "Alzheimer's" type neuropathological changes associated with a "high" probability of clinical dementia: A3, B3, C3; Cerebrovascular pathology: Cerebral amyloid angiopathy; Cerebrovascular pathology: Atherosclerotic and/or chronic hypertensive and/or diabetic vasculopathy; Absence of associated TDP-43 proteinopathy; Absence of associated Lewy body disease (alpha-synucleinopathy) |
